# Research for better health: the Panamanian priority-setting experience and the need for a new process

**DOI:** 10.1186/1478-4505-12-38

**Published:** 2014-08-12

**Authors:** Luz Isabel Romero, Cristiane Quental

**Affiliations:** 1 Universidad Santa María La Antigua, Dirección de Investigación, Apartado Postal 0819-08550, Panamá, República de Panamá; 2 Escola Nacional de Saúde Pública, Fundação Osvaldo Cruz, Rua Leopoldo Bulhões, 1480, 21041-210 Rio de Janeiro, Brasil

**Keywords:** Health research policy, Resources for research

## Abstract

**Background:**

Panama is, economically, the fastest growing country in Central America and is making efforts to improve management mechanisms for research and innovation. However, due to contextual factors, the Panamanian Health Research System is not well developed and is poorly coordinated with the Health System. Likewise, despite recent efforts to define a National Health Research Agenda, implementing this agenda and aligning it with Panamanians’ health needs remains difficult. This articles aims to review Panama’s experience in health research priority setting by analyzing the fairness of previous prioritization processes in order to promote an agreed-upon national agenda aligned with public health needs.

**Methods:**

The three health research prioritization processes performed in Panama between 2006 and 2011 were analyzed based on the guidelines established by the four “Accountability for Reasonableness” principles, namely “relevance”, “publicity”, “revision”, and “enforcement”, which provide a framework for evaluating priority-setting fairness.

**Results:**

The three health research priority-setting events performed in Panama during the reference period demonstrated a heterogeneous pattern of decision-making strategies, stakeholder group composition, and prioritization outcomes. None of the three analyzed events featured an open discussion process with the scientific community, health care providers, or civil society in order to reach consensus.

**Conclusions:**

This investigation makes evident the lack of a strategy to encourage open discussion by the multiple stakeholders and interest groups that should be involved during the priority-setting process. The analysis reveals the need for a new priority-setting exercise that validates the National Agenda, promotes its implementation by the National Secretariat for Science, Technology and Innovation in conjunction with the Ministry of Health, and empowers multiple stakeholders; such an exercise would, in turn, favor the implementation of the agenda.

## Background

Research prioritization for better health is recognized as the key factor for countries to optimize the decision-making process in order to establish their own research policies and achieve better impacts through financial investments [1]. Recently, broad concern has been raised about how to set priorities in a transparent, systematic, fair, and legitimate way in low- and middle-income countries [2-4]. The adequate fulfillment and implementation of such priorities may depend on the recognition of the agenda by actor institutions and stakeholders. Despite the fact that a universal method for priority setting for health research does not exist, several strategies and methods have been developed in order to facilitate prioritization and have been applied to promote empowerment for decision making at the national and global level [5]. A recent analysis of the prioritization experiences and methods employed by a group of 18 Latin American and Caribbean countries showed a wide variety of practices and achievements, indicating the need to use a systematic approach to develop research priorities specific to each country’s context [6].

The health research prioritization experience in Panama is recent; a National Health Research Agenda acknowledged and embraced by the Health Ministry (MINSA), the country’s governing health institution, has not been developed. Further, a systematic analysis of the practice has not been performed and prioritization has not been discussed within the scientific community. Moreover, the need to evaluate coordination between health research priorities and local realities was acknowledged during the Second Latin American Conference on Research and Innovation for Health, which took place in Panama City in November 2011 [7]. Thus, the objective of this study was to examine Panama’s experience in setting a National Health Research Agenda from a process perspective in order to improve this practice and generate agreement and fairness in future prioritization exercises.

### Local context

Panama is a Central American country of 3.8 million inhabitants and is considered the most promising economy in the region; it shows high-middle income economic performance with a gross domestic product per capita of 12,000 USD [8], the highest human development index in Central America (0.780), and the fourth highest index among Latin-American countries [9]. However, structural poverty and inequity still persist; 27.6% of the population lives below the poverty line, and 14.2% of the population lives in extreme poverty [10]. Indigenous groups compose 12.3% of the total population and are the most affected by health disparities; this disadvantage translates into high mortality and years of potential life lost due to transmissible diseases [11]. This pattern is different from that of the non-indigenous population, in which mortality from non-transmissible diseases predominates, reflecting the epidemiologic transition resulting from improved quality of life [12]. Panama does not have a universal health care system, but its implementation has been under debate for several years without a conclusion. Meanwhile, health services and social security for over 60% of the population is provided by the Caja de Seguro Social (CSS) based on contributions from employee salaries. The rest of the population is covered by a network of MINSA hospitals and health services. Together, the CSS and MINSA constitute the Public Health System of Panama; investment in this system is estimated at 1.3 billion USD, equivalent to 5% of the GDP and 14% of the government’s total expenditures [13].

As recently discussed in previous research [14], Panama does not have a structured National Health Research System (NHRS). This is related to historical factors, such as the Panama Canal construction by the US government at the beginning of the 20th century. The vector control for transmissible diseases, such as yellow fever and malaria, by William C. Gorgas in 1904, marked a success for the Panama Canal construction and the establishment of health research in the Panamanian isthmus [15]. In 1921, the Panamanian government established the Gorgas Memorial Laboratory (GML), which, under US administration, succeeded as an active tropical disease research institute. For over six decades, the GML was the only research institution in the country, and its sustainability depended in large part on the US until the 1990s [15], when the process of returning the administration and territory of the Panama Canal to Panamanian control began. The incorporation of the GML, now Gorgas Commemorative Institute of Health Studies (ICGES), into the National Health System in 1990 began the structuring of the local NHRS.

In 2003, the ICGES was restructured through Law 78 into a public, social interest entity with legal, financial, and technical autonomy, responsible for conducting and stimulating the development of national health research in coordination with MINSA [16]. However, neither MINSA nor ICGES has dictated an agenda of national priorities for health. Instead, the Plans of the National Secretariat for Science, Technology and Innovation (SENACYT) prevail. SENACYT, which is the key institution for promoting research activities, innovation, and human resources training for all knowledge areas in the country, was founded in 1992 as a joint organization of the Panamanian Presidency [17]. The institution had jurisdiction over policies and resources to accomplish its function until 2005 [17], when the National Strategic Plan for Science, Technology, and Innovation (PENCYT) became the means to execute research priorities, including the health research agenda. However, the implementation of PENCYT agendas has been compromised by the low level of local public investment in research and development activities, including the suspension of research funds during the last two years. According to recent data [18], Panama invested 0.19% of its GDP in research and development in 2010, which is four times below the average for Latin American and Caribbean countries, estimated at 0.78% of GDP. Official data indicate that Panama’s cumulative public investment in research and development, awarded through competitive grants from SENACYT between 2004 and 2012, was $18.6 million dollars. These funds have been assigned to 340 research projects in all knowledge areas, of which 63 (18.5%) correspond to health sciences [19]. The ICGES and the Institute for Scientific Research and Technology Services (INDICASAT) are the leading research institutions in the country and are the main recipients of these research funds, as indicated in Table 1.

**Table 1:** Distribution of public fund for health research during the years 2004 to 2012

**Institution**	**Project (n)**	**Investment (%)**
Gorgas Commemorative Institute of Health Studies (ICGES)	26	38.0
Institute for Scientific Research and Technology Services (INDICASAT)	20	39.9
University of Panama (UP)	6	9.3
Health Ministry (MINSA)	2	6.2
Social Security (CSS)	2	2.8
Others	7	3.8
Total	63	100

Elaborated from official data from SENACYT [19].

According to the World Health Organization, a health research prioritization process is defined as a scheme to build consensus on a set of research issues that require urgent attention [20]. A priority is defined as an element or condition judged to be more important than another, which involves an exercise in “judgment” and the use of “values” that sustain the quality of a priority [21]. Simultaneously, there is a local context and a series of criteria, such as benefits, evidence, cost-efficiency, equity, and severity, which compete in order to establish this judgment. Finally, stakeholders, who act individually in representation of pluralist society, make decisions by consensus, making an ethical framework necessary to sustain the legitimacy of the prioritization process [2,20]. This group of stakeholders includes individuals from the governmental sector, non-governmental organizations, the private sector, academia, health service providers, and healthcare recipients. The intention of this plurality is to encourage equal participation among the different sectors involved and to allow constructive debate for conflict resolution between different areas and interests [2]. An explicit prioritization process strategy and a description of follow-up plans are other key elements that guarantee the process’ transparency and legitimacy [2]; transparency refers to the extent to which it is clear how decisions were made and legitimacy refers to the moral authority of decision makers [22,23].

Daniels and Sabin [22,24] established the process of “accountability for reasonableness” (AR) with the aim to prevent errors that can be committed by legitimate authorities. AR represents the ethical framework of a legitimate prioritization process as it encourages democratic deliberation through four conditions based on justice theory: relevance, publicity, review, and enforcement. The condition of “relevance” is met if prioritization decisions are based on importance as recognized by legitimate decision makers. In order to meet this condition, careful selection of a representative group of decision makers who are responsible for the elucidation of values, criteria, methods, and information that will guide the prioritization process is necessary. The condition of “publicity” is achieved if the decisions and reasons for the prioritization are made available to encourage public debate. To meet the condition of “review”, mechanisms for the reevaluation of decisions based on new evidence should exist. Finally, to ensure a just process, the leaders must guarantee the three previous conditions are met, therefore meeting the fourth condition of “enforcement” [1,25]. Recently, the AR approach has been implemented in priority-setting for health care services [26,27] and health research [28,29], relying on the belief that fair, deliberative procedures yield acceptable results [26].

The principal objective of establishing research priorities for health at the domestic or international level is to align investments with the population’s health needs in an efficient way to improve health and quality of life. However, prioritization represents one of the greatest challenges confronted by decision makers, particularly in less developed countries where there are obstacles, including limited economic resources, insufficient official health indicators, socioeconomic inequity, political instability, and inefficient health institutions and systems [1,30]. At the same time, one of the largest challenges is the lack of equal participation among decision makers and the shortage of systematic strategies for adequate prioritization processes [31]. As a result, at times, countries set priorities based on historical precedents or by forming *ad hoc* committees [31]. These prioritization practices frequently generate results that are not in line with reality or that are influenced by the preferences and scientific autonomy of researchers [31].

Systematic prioritization process experiences of low- and middle-income countries in Africa, Asia, and Latin America have been documented [2,5,6]. Authors report high heterogeneity among the different countries examined in areas such as the context, methods used, criteria included, information used, involvement of different stakeholder groups, results obtained, and prioritization processes [2]. In the Latin American context, the leadership of Brazil has been recognized for the transparency of its processes, the inclusion of broad stakeholder groups, and the maturity of its health research system. Brazil’s prioritization is based on a high degree of public consultation throughout the process, which was led by the Ministry of Health in 2003 and followed the Combined Approach Matrix methodology. The first draft of the agenda was written by a group of 510 professionals from five different regions [32], and it was available for public comment over 45 days on the Ministry of Health’s official website. A series of municipal, state, and national conferences constituted the preparatory phase with the community before the agenda was approved during the Second National Conference on Science, Technology, and Innovation in 2004. The event included the participation of 431 conference delegates and 213 observers and invited guests. Six basic principles guided the construction of the agenda: 1) improve public health in the short, medium, and long term; 2) eliminate all forms of inequality and discrimination; 3) respect the life and dignity of people; 4) ensure the implementation of high ethical and gender standards in research; 5) respect methodological and philosophical pluralism; and 6) strive for social inclusion and environmental respect [32]. Thus, if evaluated with the AR principles in mind, the described country experience illustrates the establishment of a comprehensive national agenda with a high level of legitimacy based on deliberative practices for consensus building and transparency.

## Methods

This qualitative study was based on the analysis of agenda setting for health research performed in Panama between 2006 and 2011, after science and technology management mechanisms were established in the country. The three prioritization events identified were documented in key official documents from SENACYT, available via the transparency module of its official webpage (http://www.senacyt.gob.pa): the PENCYT 2006–2010 [17], the PENCYT 2010–2014 [33], and the “*Final report on the inter-sectoral and inter-institutional workshop on health research policies and priorities*” [34]. The conceptual framework for this analysis was based on the four AR principles of “relevance”, “publicity”, “revision”, and “enforcement” [22-24]. Table 2 provides a summary of this methodology and its application to this study.

**Table 2:** Description of the “Accountability for Reasonableness” conditions

**Condition**	*As described	**As applied in this study
Relevance	Rationales must rest on evidence, reasons, and principles that all fair-minded parties can agree	Assessing the use of evidence, principles or criteria to stand the decisions taken during the priority setting process
Publicity	Decisions and their rationales must be publicly accessible	Assessing the access of the defined priority agenda and its public discussion previous to its official approval
Revision	Mechanism for revising decisions in light of further evidence or arguments	Assessing the agenda reevaluation and actualization following external or internal discussion/evaluation
Enforcement	A process to ensure that the first three conditions are met	Assessing the leaders engagement for the accomplishment of the previous conditions

*From references [22-24].

**As described in Methods.

## Results

### Priority-setting processes description

Panama has no exclusive policy regarding research and innovation for health. Instead, the PENCYT acts as SENACYT’s official plan and operative instrument. The PENCYT is established every five years and is composed of seven sectoral plans, including one on health. Three PENCYTs have been written since SENACYT was established in 1992. The first one corresponded to the years 1998 to 2000 [17]. During the following 6 years, the country operated without an official plan until the PENCYT 2006–2010 was officially approved [17]. The current PENCYT corresponds to the 2010–2014 period [33]. To implement the plans, SENACYT established the science and technology management mechanisms in 2005. Therefore, for the purposes of this study, only the three prioritization exercises performed after 2005 were analyzed.

Two prioritization processes took place to establish the PENCYT 2006–2010. During the first process [Priority-setting I], the “Sectoral plan for health research and innovation” was elaborated by an *ad hoc* Health Commission, which consisted of six researchers from only two institutions (ICGES and Children’s Hospital) (Table 3) [17]. Once placed within the context of the country’s health situation using health data, the Commission proceeded with the identification of priorities for the transformation of the national public health situation, a vision that guided the construction of nine themes, as illustrated in Table 4. The plan, which became official in February of 2007, did not pass through a consultation process with the scientific community, nor was it made available for public comment or consultation before being approved. During the same year, PENCYT 2006–2010 underwent an external mid-term review process. This review recognized that a systematic prioritization exercise was needed for health research to complement the Priority-setting I process.

**Table 3:** Comparative analysis according to the criteria of Accountability for Reasonableness (AR) between different health research prioritization processes in Panama, 2006 to 2012

	**Decision makers**		**Criteria**			
**Plan**	** Committee**	** Institution (n)**	**Relevance**	** Publicity**	** Review**	** Reinforcement**
Priority-setting I:	*Ad Hoc* of 6 researchers	ICGES (5) HN (1)	Present	Not present	Follow-up review, present	Review was not incorporated
PENCYT 2006–2010						
Priority-setting II:	Pluralist participation of 65 actors from the public and private sector, government, academia and ST&I management	MINSA (25), ICGES (13), UP (7), HST (5), COHRED (1),	Present	Not present	Not present	Partially incorporated into PENCYT 2010–2014
Inter-sectoral and inter-institutional workshop on policies and priorities in health research						
		SENACYT (1), CSS (1), Comptroller (2),				
		ANAM (1), Private (1), INDICASAT (1),				
		HN (2), MIDA (4), NGO (1)				
Priority –setting III:	*Ad Hoc* committee of 13 actors and 3 collaborators	MINSA (4), ICGES (2), CSS (2),	Present	Not present	Present	Not present
PENCYT 2010-2014		UP (2), NGO (1)				
		STRI (1), HN (1)				
		UP (1), INDICASAT (1), Government (1)				

ANAM, Autoridad Nacional del Ambiente (National Environmental Authority); COHRED, Council on Health Research for Development; CSS, Caja del Seguro Social; HN, Hospital del Niño (Children’s Hospital); HST, Hospital Santo Tomás; ICGES, Gorgas Commemorative Institute of Health Studies; INDICASAT, Institute for Scientific Research and Technology Services; MIDA, Ministerio de Desarrollo Agropecuario (Ministry of Agricultural Development); MINSA, Health Ministry; NGO, Non-governmental organization; PENCYT, National Strategic Plan for Science, Technology, and Innovation; SENACYT, National Secretariat for Science, Technology and Innovation; STRI, Smithsonian Tropical Research Institute; UP, Universidad de Panamá.

Source: Elaborated from official documents available from SENACYT’s webpage [17,33,34].

**Table 4:** Structure of the agenda of priorities generated by the health research prioritization processes conducted in Panama, 2006 to 2015

**Plan/Policy**	**Structure and priorities**
Sectoral plan of research and innovation in health- PENCYT 2006–2010 [17] (Priority-setting I)	Structured into nine Health Themes:
	1- Consolidation of health research as a generator of evidence for decision making.
	2- Reinforcement of research in prioritized disease and mortality themes (non-transmissible diseases).
	3- Reinforcement of research for the prevention and control of transmissible diseases.
	4- Reinforcement of research in health management, public health, and service provision.
	5- Utilization of the System to achieve better impact from health actions.
	6- Determination of social factors and risk factors of disease.
	7- Development of a health research system.
	8- Development of research in technology evaluation.
	9- Strengthen the capacity of ICGES’s leadership.
Inter-sectoral and inter-institutional workshop on policies and priorities in health research 2007 [33] (Priority-setting II)	Structured into six General Areas:
	1- Health and environment: water contamination/exposure to transmissible disease vectors.
	2- Behavior and lifestyle: nutrition/problems linked to exposure to social risks.
	3- Education and citizen participation: health education/awareness of rights and responsibilities in health.
	4- Disease burden and mortality: transmissible diseases/emergent and re-emergent diseases.
	5- Health services: organization of services/quality of services.
	6- Inequality in health: inequality in resources in services offered/distribution of mortality, life expectancy, YPLL, birth weight, median school-age, height, and psychomotor development.
National ST&I Program for the Development of Biosciences and Health Sciences/PENCYT 2010–2014 [34] (Priority-setting III)	Complex structure simultaneously applied to biosciences and health sciences:
	1- Priority actions for human resources reinforcement.
	2- Priority areas in bioscience and health science training.
	3- Priority actions at the level of research and development in biosciences and health sciences.
	4- Priority thematic areas in R&D in biosciences and health sciences:
	a) Prioritization workshop areas (COHRED-SENACYT 2007) (see previous plan).
	b) Research priorities associated with specific diseases in the short, medium, and long term.
	c) Other thematic priorities in research and health determinants.
	d) Priorities at the level of innovation (national registry system of research projects and others).

COHRED, Council on Health Research for Development; ICGES, Gorgas Commemorative Institute of Health Studies; SENACYT, National Secretariat for Science, Technology and Innovation; PENCYT, National Strategic Plan for Science, Technology, and Innovation; YPLL, Years of potential life lost.

Source: Elaborated from official documents available from SENACYT’s webpage [17,33,34].

According to the AR framework, Priority-setting I met the “relevance” principle as epidemiological evidence was analyzed to determine the relevance of problems to be discussed, but the process failed to satisfy the “publicity” criterion when PENCYT 2006–2010 was approved without community consultation. The “revision” principle was also met as the plan was submitted to external evaluation; however, the process failed to meet the “enforcement” criterion, which requires leadership engagement to meet the three previous conditions.

The second prioritization process [Priority-setting II] was performed following PENCYT’s external mid-term review process, which recognized that a systematic prioritization exercise was needed. The prioritization event was organized by SENACYT during the “Final report on the inter-sectoral and inter-institutional workshop on health research policies and priorities”, which took place in November 2007 [34]. In this case, the priority-setting exercise was led by ICGES and was conducted in consultation with the Council on Health Research for Development (COHRED) [34], a global, non-profit organization that supports the development of research systems in low- and middle-income countries. The process was based on the Hanlon method [34], a quantitative tool designed to rank selected health problems according to specific criteria in order to reach consensus among a wide participation of actors [35]. Thus, in Priority-setting II, the work group comprised 65 participants including researchers, managers, academics, decision makers, and members of civil society from different regions of the country (Table 3). The participants were divided into six working groups coordinated by two facilitators. To initiate the prioritization, the most important health issues were previously categorized into six general areas as indicated in Table 4. For each area, five specific problems were discussed and subjected to four evaluation criteria using a quantitative scale: magnitude (A), severity (B), cost-efficiency (C), and feasibility of intervention (D). The results obtained were incorporated into a single prioritization matrix and then tabulated using a formula [priority = (A + B) × C × D] to obtain a final quantitative value, as detailed by the documentation generated during the prioritization process [34]. The results were then ranked from highest to lowest numerical value to obtain an order of prioritized problems (Table 4). However, the results of this process were not incorporated into the PENCYT, nor were they assimilated by MINSA at the time.

When evaluated according to the AR criteria, Priority-setting II met the “relevance” principle because epidemiological evidence was reviewed prior to the discussion. In addition, a well-defined, systematic prioritization method with clear selection criteria was applied and its strategy was explicit. However, with regards to “publicity”, this criterion was not met; despite efforts to ensure plurality of the decision-making group to promote agreement in the absence of public consultation, an imbalance in representation was observed. For instance, the CSS, which provides public health care services to over 60% of the population, was underrepresented with only one participant (1/65), compared to 25 (25/65) participants from MINSA [34]. Another important actor institution that was not represented was the National Oncologic Institute; this institution provides health services to all Panamanian patients with cancer and malignancies, which are the second leading cause of death in the country [36]. As there was no “revision” of decisions in light of new evidence documented at the time, and “enforcement” of all the principles was not observed, these criteria were not met in Priority-setting II.

The formulation of the current PENCYT 2010–2014 presented a new opportunity for organizing the agenda [Priority-setting III] [33]. In contrast to the previous PENCYT, in which biosciences and health were maintained in separate plans, the current PENCYT 2010–2014 integrated both areas into the “National Program of Science, Technology and Innovation for the Development of Biosciences and Health Sciences”. The Plan’s priorities were determined by an *ad hoc* Sectoral Commission made up of 13 actors and 3 collaborators led by a coordinator from the University of Panama (Table 3). The group of stakeholders from the Sectoral Commission with a decision-making function was composed mostly of researchers representing public research institutions (n = 3), health care facilities (n = 1), academia (n = 2), non-governmental organizations (n = 1), and a non-Panamanian research institution (n = 1). Minor stakeholder representation corresponded to policy makers from health (n = 4) and science and technology (n = 1) governmental institutions.

Once the evidence was reviewed and an analysis of the sector’s strengths, weaknesses, opportunities, and threats was completed, the Commission produced a series of priorities grouped into different categories with a complex structure. As illustrated in Table 4, the first four categories were organized as actions and areas for scientific research strengthening and development in both health and biosciences (biology, biodiversity, ecology, and environment). The identified problems from Priority-setting II were then incorporated into the priority list. This was followed by formulating a new category of “research priorities linked to specific diseases”, which were separated into short-, medium-, and long-term goals. Then, “other thematic research priorities” were added. Finally, two “priorities at the level of innovation” were incorporated: 1) a national registry system of researchers and research projects and 2) access to scientific publication databases. A series of action lines and strategies were dictated for achieving these priorities and when finished, the PENCYT 2010–2014 passed through an approval process with the Cabinet Council in December 2010. The Plan is available on SENACYT’s official webpage (http://www.senacyt.gob.pa) but did not pass through a consultation process with the scientific community. This plan was recently subjected to an external evaluation of medium-term validity to assess its implementation.

According to the AR approach, Priority-setting III met the “relevance” principle as health evidence was widely analyzed before the prioritization exercise. The “revision” criterion was partially met; the implementation of the plan but not the selection of themes during the prioritization process was reviewed by an external commission. On the other hand, the “publicity” principle was not satisfied as decisions were made in a closed event by a small committee without public discussion. This lack of open participation also had a negative impact on the principle of “enforcement”, which reassures that the three previous principles have been met.

## Discussion

Currently, there is an increased emphasis on health research prioritization in a transparent and legitimate way [5], particularly because important economic decisions regarding public funding are derived from prioritization outcomes. However, prioritization is difficult to perform as the process depends on the application of a series of criteria and values interpreted by many different actors according to the socioeconomic and political context where the process occurs. Based on these levels of complexity and responsibility, the objective of the present analysis is to promote critical evaluation of the health research priority-setting experience in Panama to optimize decision making in order to form an agreed-upon health research agenda in accordance with public health needs.

The present study analyzed Panama’s most recent prioritization processes based on the AR conditions [22,24] with the objective to identify good practices and opportunities for improvement. The short history of the NHRS in Panama reveals limited experience in systematic decision making for health research prioritization, particularly in a participatory and decentralized practice [15,37]. The results of the prioritization processes presented in this analysis reflect the evolving health research situation in the country during recent years; from 1921 to only a decade ago, research was sustained predominantly by one institution focused primarily on tropical, transmissible diseases, the ICGES. The second institution in the country dedicated to biomedical and environmental research, INDICASAT, was established in 2002. Only in recent years have research groups been organized at both institutes and competitive local scientific production started. On the other hand, clinical research has been sustained by a few research groups in some hospitals and health centers within the country. In a number of cases, this research is related to the application of a pre-defined protocol for multi-center drug and vaccine clinical trials, which are often not considered local scientific production. The University of Panama, the country’s main university, has few research groups in the basic sciences and has maintained low and sustained productivity in the areas of pharmacology, parasitology, chemistry, and molecular biology [14]. Based on the present analysis, one can understand why a balanced representation of actors was not observed in Priority-setting I and II. The intention to overcome this limitation during Priority-setting II improved the plurality factor; this exercise was based on a wide participation of stakeholders in the fields of health care and health research and from civil society; however, balanced participation among the health system actors was not achieved. The health care participant groups were not equally represented: MINSA (25/65) vs*.* CSS (1/65). The CSS, the underrepresented institution, provides health assistance to over 60% of the Panamanian population and takes on the high cost of non-transmissible diseases, the main cause of death in the country. The lack of equal participation by decision makers has been identified by COHRED as a source of potential errors that can influence the prioritization process and its results [38]. To this problem we can add the potential risk for a lack of agenda receptivity and ownership by stakeholders and actor institutions.

The formulation of the PENCYT 2010–2014 provided a new opportunity to overcome the equal representation challenges of the previous processes. However, the reduced size of the commission and the composition of actors, including only researchers (n = 7) and policy makers (n = 5), limited wider participation by other actors; decisions were made based on the *ad hoc* commission’s perspective in a closed event.

Among the risks encountered when prioritization processes lack consultation and consensus by a wide group of actors is that the prioritization results are not in accordance with the public health plans that the country needs. The limited representation of health care providers and health service users in the discussion may have caused service quality and health equity to be overlooked as health issues needing attention. In addition to the need to promote participation by more actors in the decision-making process, there is also a need to improve agreement on health problems relevant to the general population. Public consultation to reach consensus has been a mechanism through which other Latin American countries, such as Brazil, Colombia, and Peru, have established health priority agendas and legitimized the decision-making process [4,6].

The theme of health research in the plans analyzed in the present study deserves clear discussion as it overlaps with the area of biosciences within the current PENCYT 2010–2014. In this case, health research has been categorized as a biological discipline, which maintains the biotechnology paradigm as the primary axis for establishing the agenda. On the other hand, we must consider that health frequently overlaps with the social, environmental, and agricultural sectors [4], among others. As a result of the simultaneous approach to bioscience and health, the priorities written in the current plan are ambiguous, the structure is difficult to understand, and its implementation by health institutions is difficult. These factors represent an obstacle to the basic function of priority setting, which is to identify important health issues and mobilize the resources needed to address them. In the ideal case, the agenda represents agreed-upon topics or health problems resulting from a prioritization process and becomes the policy instrument that mobilizes the health research system through specific calls for proposals and resource allocations [39]. Consequently, a national research portfolio aligned with population health needs to generate evidence for health improvement and country development is the overall objective of health research.

Therefore, establishing a national agenda using public health and equity lenses to address the problems most affecting the Panamanian population, such as non-communicable diseases, population aging, and deaths due to accidents and violence, as well as health promotion and disease prevention, remains the objective for the next prioritization exercise. Ensuring fairness of the priority-setting process by including a wide range of stakeholders and an open discussion is the means by which a comprehensive plan that is recognized by different interest groups can be formed. Last but not least, a unified vision of health and science and technology government institutions that prioritizes and guarantees investment in health research is the overarching goal that will assure implementation of a national health research agenda.

## Conclusions

This analysis reveals the need to rethink a health research prioritization strategy in which wide participation from stakeholders working towards consensus at the national level is included. With this, the prioritization process’ legitimacy will be improved, the health research policy will be strengthened, and the agenda can be structured to be aligned with public health needs in order to improve the population’s health and quality of life.

## Abbreviations

AR: Accountability for reasonableness; COHRED: Council on Health Research for Development; CSS: Caja del Seguro Social; GML: Gorgas Memorial Laboratory; ICGES: Gorgas Commemorative Institute of Health Studies; INDICASAT: Institute for Scientific Research and Technology Services; MINSA: Health Ministry; NHRS: National Health Research System; PENCYT: National Strategic Plan for Science, Technology, and Innovation; SENACYT: National Secretariat for Science, Technology and Innovation; STRI: Smithsonian Tropical Research Institute.

## Competing interests

The authors declare that they have no competing interests.

## Authors’ contributions

LR was responsible for the concept of the study, collected and analyzed the relevant data, and drafted the manuscript. CQ contributed to the overall concept of the study, provided guidance on the paper’s intellectual content, and refined the original version of the manuscript. Both authors read and approved the final manuscript.

## Authors’ information

LR is currently the Research Director of Universidad Santa Maria la Antigua of Panama. Previously, the author served as staff scientist and Acting Director of INDICASAT as well as member of the executive directors committee at SENACYT during the establishment of the national science and technology operative management mechanisms. LR holds a PhD in public health, graduated at the National School of Public Health – FIOCRUZ, Rio de Janeiro, Brazil.

CQ has been a Professor in the Department of Planning and Analysis, at the National School of Public Health – FIOCRUZ, Rio de Janeiro, Brazil. She is an economist and her expertise is in the field of Science, Technology and Innovation analysis, and she mentored LR in her doctoral thesis on public health. All authors read and approved the final manuscript.
